# Experiment research of focused ultrasound combined with drug and microbubble for treatment of central nervous system leukemia

**DOI:** 10.18632/oncotarget.23521

**Published:** 2017-12-20

**Authors:** Xiao-Ping Xi, Yu-Jin Zong, Yan-Hong Ji, Bing Wang, Hua-Sheng Liu

**Affiliations:** ^1^ Department of Hematology, The First Affiliated Hospital of Xi’an Jiaotong University, Xi’an 710061, China; ^2^ Department of Hematology, The First Affiliated Hospital, and College of Clinical Medicine of Henan University of Science and Technology, Luoyang 471003, China; ^3^ The Key Laboratory of Biomedical Information Engineering of Ministry of Education, Department of Biomedical Engineering, School of Life Science and Technology, Xi’an Jiaotong University, Xi’an 710049, China; ^4^ Department of Immunology, Xi’an Jiaotong University, Xi’an 710061, China; ^5^ Department of Pathology, Xi’an Jiaotong University, Xi’an 710061, China

**Keywords:** CNSL, ultrasound, Sonovue^®^, Ara-c

## Abstract

It has been shown that low frequency ultrasound in the presence of microbubble can effectively open the blood brain barrier (BBB) to allow the drugs to be delivered into the brain with an increased concentration. We aim to apply this method to increase the efficacy of Cytarabine (Ara-c) to treat central nervous system leukemia (CNSL). In the present study, we validated this ultrasound contrast agent Sonovue^®^ targeting treatment via *in vivo* and *in vitro* experiments. The results showed that Sonovue^®^ combined with Cytarabine could significantly inhibit K562 cell (chronic myeloid leukemia cell line) proliferation. In the animal experiments, it has been shown that high dose Ara-c chemotherapy could prevent and cure CNSL effectively and the drug concentration in the brain was much higher compared with low dose Ara-c chemotherapy group. We certified that under ultrasound exposure Sonovue^®^ combined with low dose Cytarabine achieved an effective drug concentration in the rat brain, and brain tissue had no significant damage. Further animal experiments will be conducted to confirm these results in a leukemia animal model, considering the blood brain barrier is destroyed at different levels by leukemia cells. We hope this method will reduce the side effects of high-dose Cytarabine and improve the clinically high recurrence and poor prognosis of the central nervous system leukemia.

## INTRODUCTION

Central nervous system leukemia (CNSL) is a fatal leukemia complication companied with clinical symptoms in nervous system due to the infiltration of leukemia cells into duramater, spinal cord, brain parenchyma and nervous system [1]. CNSL is an indispensable factor for the treatment of acute leukemia and other hematological malignancies [2]. Although advanced methods have been developed to delay the disease progression such as lumbar puncture intrathecal injection, high-dose chemotherapy, cerebral radiation and hematopoietic stem cell transplantation therapies [3], the cure rate remains low [4]. CNSL has a tendency to relapse and exhibit refractoriness, thus fails to achieve the long-term goal, which is to reduce recurrence, to prolong survival and to improve life quality. Therefore, it's necessary to find a new, safe and effective treatment method to prevent and control CNSL or other hematological malignancies infiltrating central nervous system.

The blood brain barrier (BBB) functions to inhibit the delivery of an amount of agents to the brain, including 100% of large-molecule neurotherapeutics and more than 98% of small-molecule drugs [5]. Overcoming the difficulty in delivering therapeutic agents to brain presents a major challenge for the treatment of CNSL. Blood brain barrier may also participate in forming and maintaining the CNS immune evasion [6], which is the basis of recurrence that leukemia cells and other tumor cells hide in the “shelter-central nervous system” to “escape” the damage of chemotherapy drugs [6]. In clinic, doctors often use a high drug dose to achieve enough drug concentration in brain, which also increases the toxicity risk [7]. Therefore, for CNSL or other hematological malignancies central nervous system infiltration treatment, a new drug delivery system by which the drug can effectively pass through the BBB is indispensable.

Ultrasound-targeted microbubble destruction (UTMD) [8] refers to the microbubbles fracture that arouses cavitation effect [9] under ultrasound exposure with different intensities at a specific site, in turn the macromolecular substance can pass through the cell membrane due to sonoporation and be caught by cells [8, 10–12]. This is a potential method for targeted drug delivery. It has been proved that high dose Ara-c chemotherapy can prevent and cure CNSL effectively [13] and the drug concentration in the brain is much higher than that in low dose Ara-c cheotherapy. This study aims to investigate the treatment effect of ultrasound microbubble carrying low dose Cytarabine in order to build the basic therapeutic method to treat the central nervous system leukemia.

## RESULTS

### Selection of ultrasonic exposure parameters, Sonovue^®^ concentration and Cytarabine concentration

**Figure 1:**
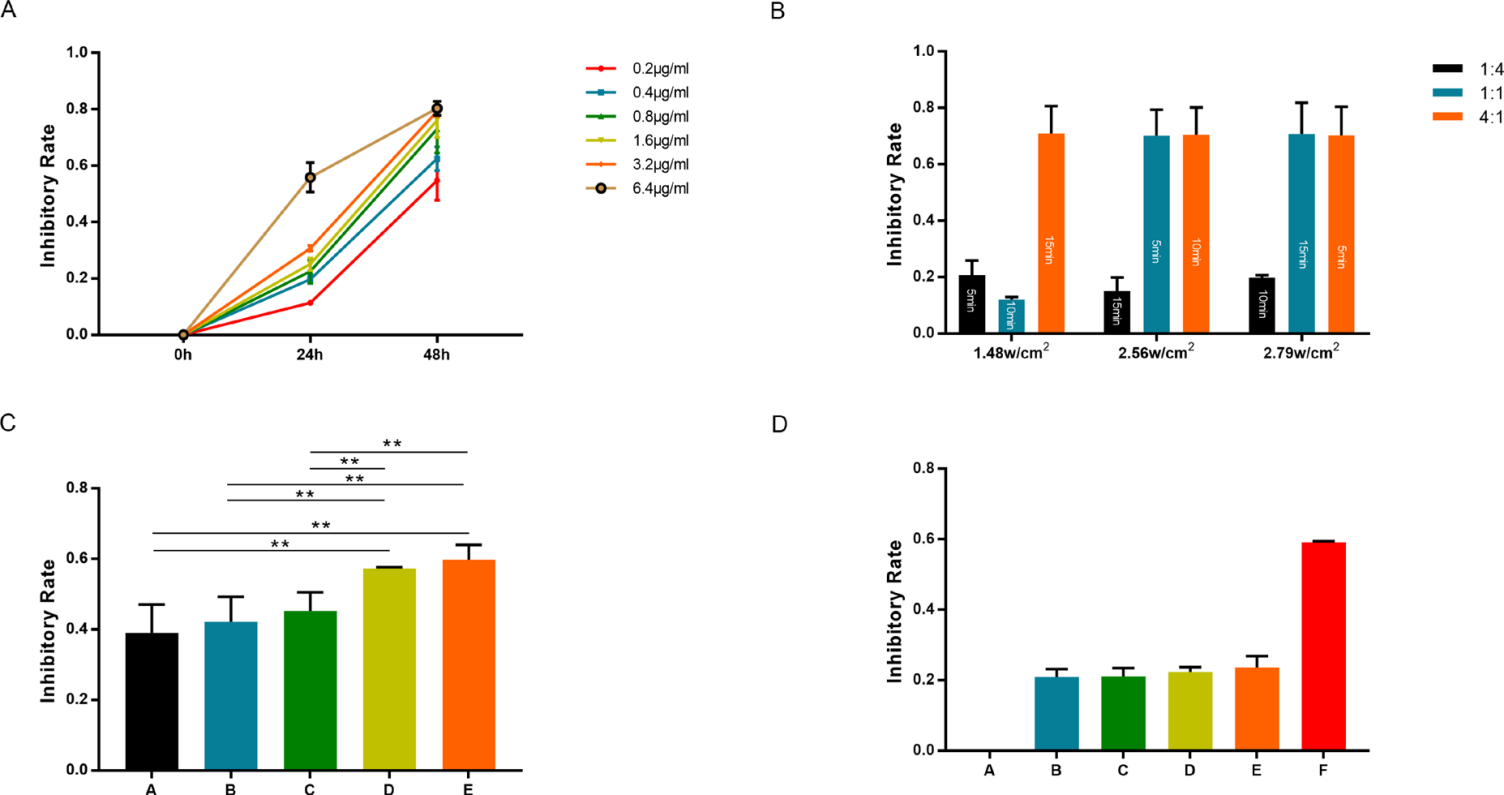
k562 cell proliferation inhibition rate determined by MTT with different Cytarabine concentration: 0.2 ug/ml–6.4 ug/ml (**A**); under different ultrasonic parameters in orthogonal experiment: date shows in Table 1 (**B**); with different Sonovue^®^ concentration (**C**, column A, B, C, D and E means Sonovue^®^ concentration of 0.17 mg/ml, 0.85 mg/ml, 1.70 mg/ml, 3.40 mg/ml and 6.80 mg/ml); in different Ultrasound Sonovue^®^ with Cytarabine combination groups (**D**, column A, B, ……, F means: A: Blank group; B: Cytarabine group; C: Ultrasound Sonovue^®^ group; D: Sonovue^®^ drug group; E: Ultrasonic drug group, and F: Ultrasound Sonovue^®^ drug group). ^*^denotes *p <* 0.05 ^**^denotes *p* < 0.01 ^***^denotes *p <* 0.001.

(1) Cytarabine with a total concentration in the range of 0.2 ug/ml–6.4 ug/ml could obviously inhibit the growth of k562 cells at 24 and 48 hours after intervention, as shown in Figure 1. Among them, the concentration of 3ug/ml less than the concentration of IC50 at 24 hours after intervention was chosen to be used in the following experiments.

**Table 1:** Ultrasonic parameters of orthogonal design of experiment at different level

Level	Sound intensity (w/cm^2^)	duty cycle	Time
1	1.48	1:4	5 min
2	2.56	1:1	10 min
3	2.79	4:1	15 min

(2) Each adjustable parameter of the ultrasonic apparatus including three levels are listed in Table 1. According to the results of k562 cells proliferation inhibition rate after 24 hours under different ultrasonic parameters in orthogonal experiment, the best ultrasonic parameter that leads to inhibition rate <50% and takes less time was chosen (Figure 1B).

(3) According to the analysis results of different ultrasonic parameters on the inhibition of k562 cells, the order of the three factors that affect the inhibition rate is: duty ratio > time > sound intensity (Table 2). Ultimately we chose the following ultrasonic parameters sound intensity 1.48 w/cm^2^, duty ratio 1:4, time 5 min for further experiments.

**Table 2:** Range analysis results

Kjm/kjm	A (sound intensity)	B (Duty cycle)	C (Time)
K1	103.60	55.68	161.03
K2	155.70	152.87	102.32
K3	160.88	211.63	156.83
k1	34.53	18.56	53.68
k2	51.90	50.96	34.11
k3	53.63	87.52	52.28
Range	19.10	68.96	19.57

(Footnotes: Kjm for the first j column m level indicators and the corresponding experiment, such as KA1 refers to sound intensity level 11.48 w/cm^2^ of the sum of three groups of inhibition rate, Kjm for Kjm average, poor for a maximum of Kjm minus the minimum).

(4) Inhibition rate of k562 cells resulting from different Sonovue^®^ concentration (0.17 mg/ml–6.8 mg/ml) combined with Cytarabine is shown in Figure 1C. Considering the economic reason, chose final Sonovue^®^ concentration of 1.7 mg/ml into the next experiment.

### Low-intensity pulsed ultrasound (LIPUS) with Cytarabine and Sonovue^®^ inhibit k562 cells proliferation

**Figure 2:**
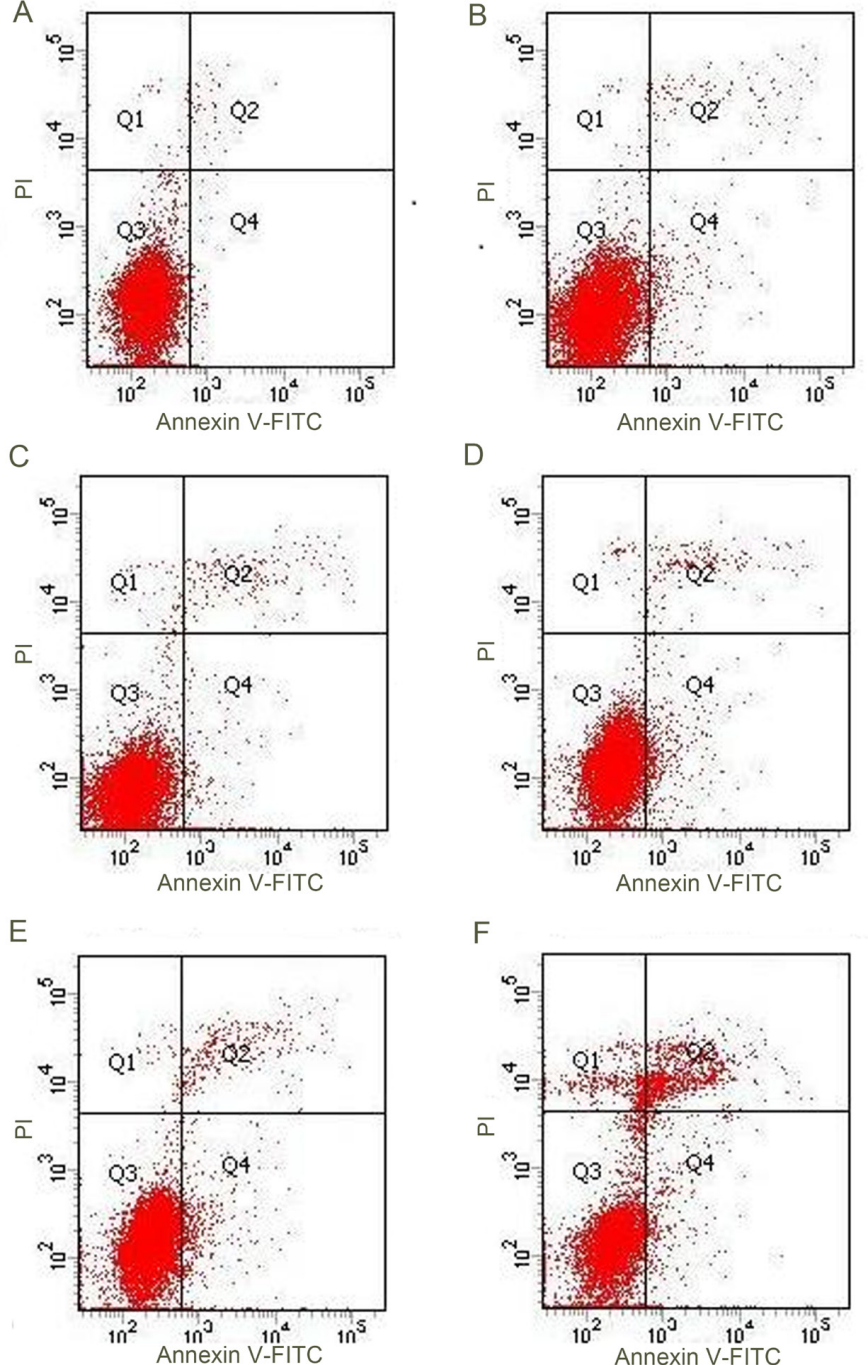
Ultrasound Sonovue^®^ combination of drugs on k562 FCM results (**A**) Blank group; (**B**) Cytarabine group; (**C**) Ultrasound Sonovue^®^ group ; (**D**) Sonovue^®^ drug group ; (**E**) Ultrasonic drug group; (**F**) Ultrasound Sonovue^®^ drug group.

**Figure 3:**
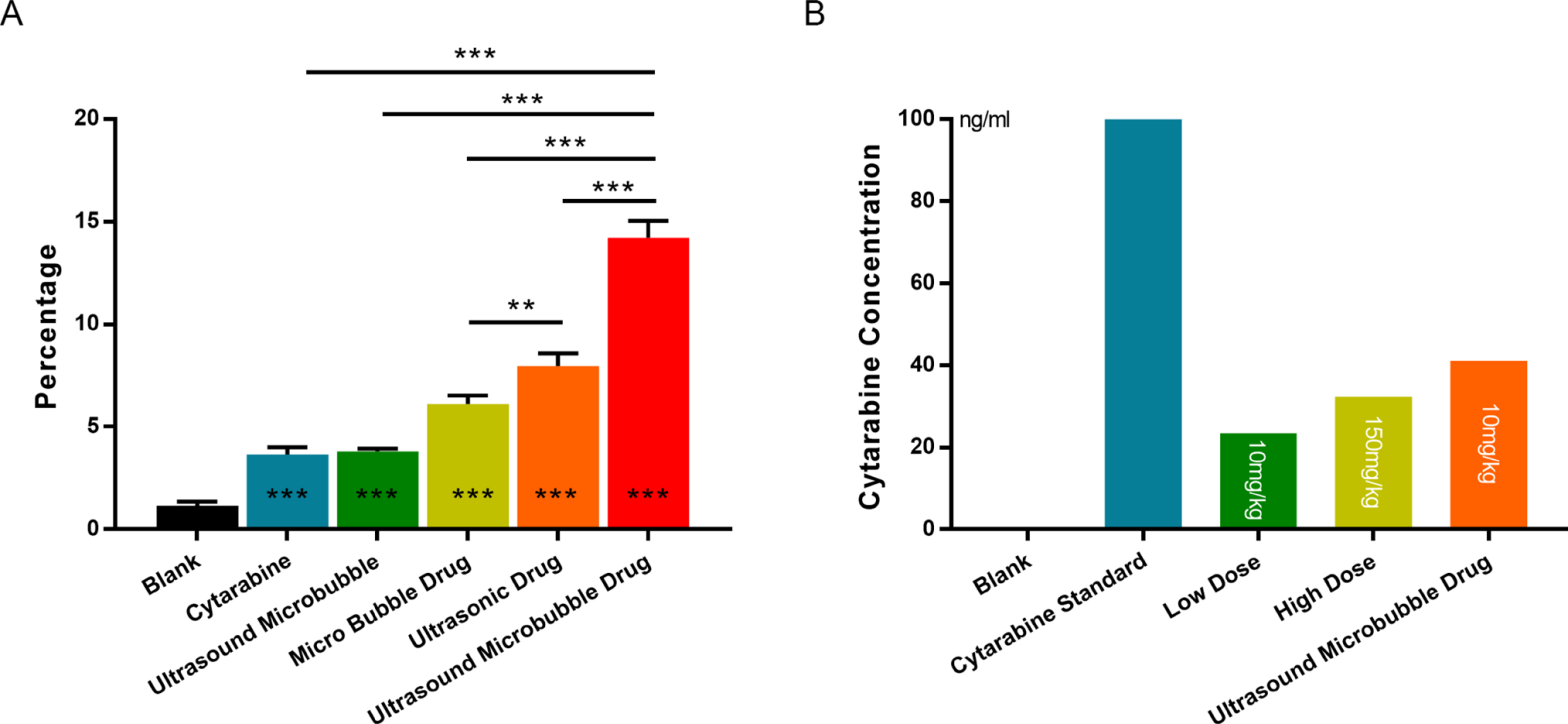
(**A**) Flow cytometer detection ultrasound Sonovue^®^ combination of drugs on k562 cell apoptosis rate: different groups and results corresponding with the results of FCM, Figure 2. (**B**) Brain tissue Cytarabine concentration measured by HPLC/MS: different dose Cytarabine with or without microbubble. ^*^denotes *p <* 0.05 ^**^denotes *p <* 0.01 ^***^denotes *p <* 0.001.

**Figure 4:**
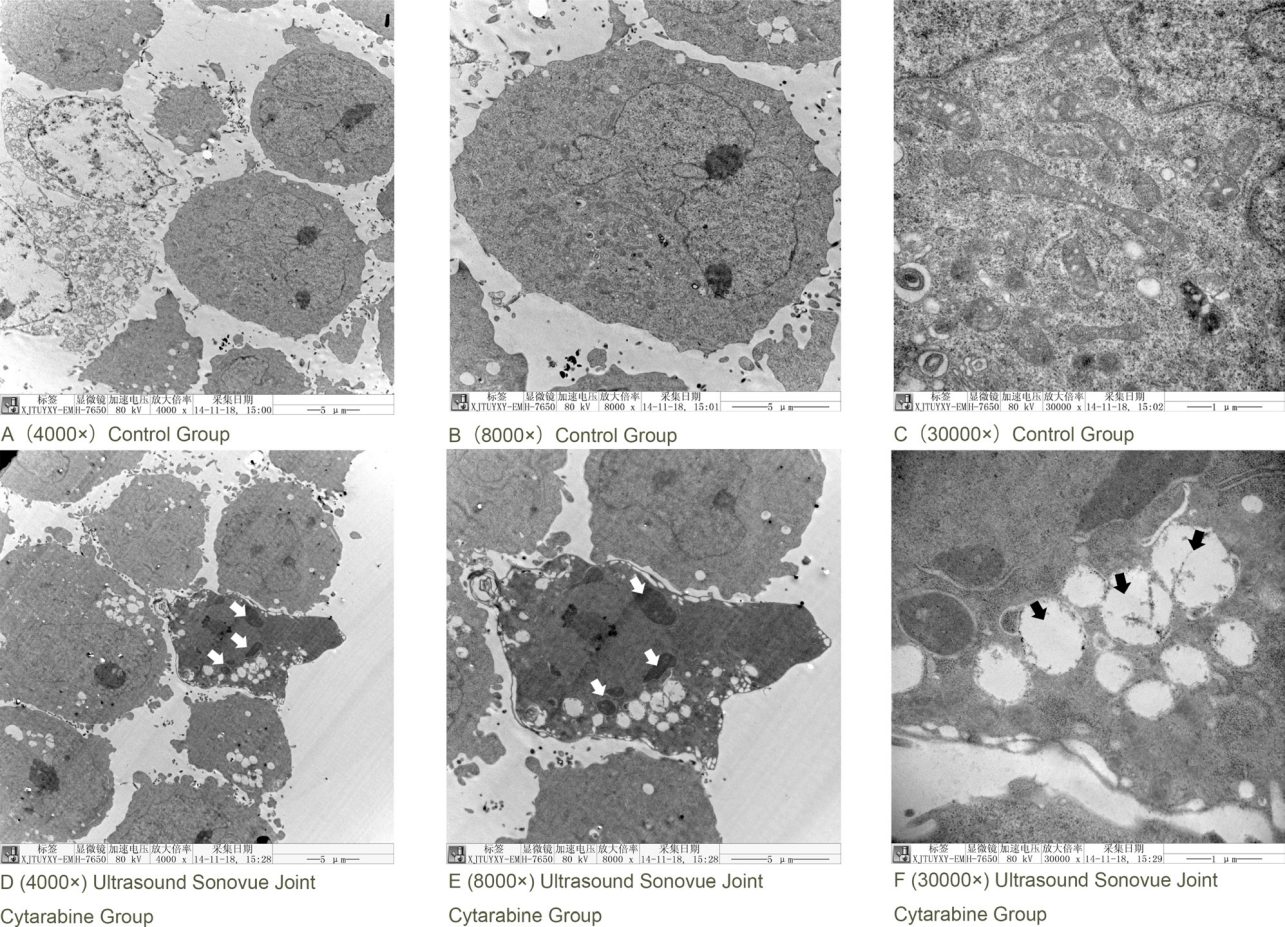
Ultrasound Sonovue^®^ joint Cytarabine intervention in k562 cells in electron microscope (**A**) Blank Group 4000×; (**B**) Blank Group 8000×; (**C**) Blank Group 30000×; (**D**) Ultrasound Sonovue^®^ combined with drug group 4000×; (**E**) Ultrasound Sonovue^®^ combined with drug group 8000×; (**F**) Ultrasound Sonovue^®^ combined with drug group 30000×.

(1) Enhanced apoptosis effect on k562 cells was detected at 24 hours after intervention with ultrasound combined with Sonovue^®^ and Cytarabine, and the effect was stronger than that in the groups with ultrasound, Sonovue^®^, Cytarabine individually used or combination of two, as shown in Figures 1D, 2 and 3A.

(2) As shown in Figure 4, the cellular morphology of untreated K562 cells are round or irregular form, the large nucleus has high nuclear/cytoplasmic ratios, and the chromatin distributed evenly in the nucleus; mitochondria of these cells are plentiful, with a circular or pole form, explicit structure, visible mitochondrial crista (Figure 4A–4C). The typical apoptotic changes were found in Ultrasound Sonovue^®^ combined with drug group: the cell is irregular, hyperchromatic, chromatin nuclear fragmentation, chromatin is crescent edge set, or agglutination into pieces; and visible mitochondria swell, cristae mitochondria structure fuzzy, glycogen accumulation, a large number of vacuoles in cells forming. (Figure 4D–4F).

### Blood brain barrier (BBB) opening induced by Low-intensity pulsed ultrasound (LIPUS) with Sonovue^®^

**Figure 5:**
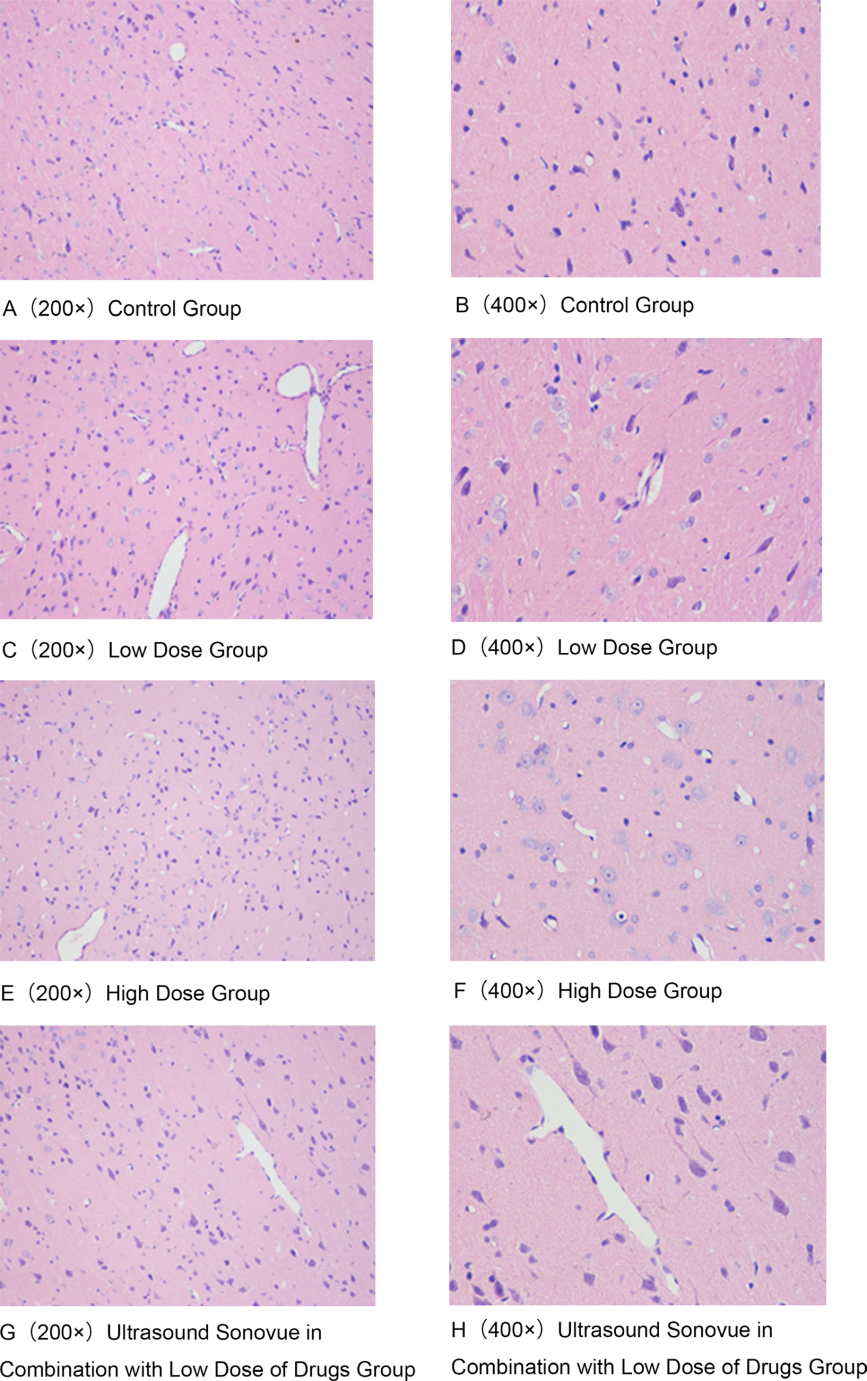
Ultrasound Sonovue^®^ combination of Cytarabine on brain HE dyeing results (Different groups indicated the RBC seepage situation has no difference.) (**A**) Blank Group 200×; (**B**) Blank Group 400×; (**C**) Low Dose Group 200×; (**D**) Low Dose Group 400×; (**E**) High Dose Group200×; (**F**) High Dose Group 400×; (**G**) Ultrasound Sonovue^®^ combined with Low Dose Drug Group 200×; (**H**) Ultrasound Sonovue^®^ combined with Low Dose Drug Group 400×.

In consideration of RBC seepage situation as the main basis for vascular and tissue damage, the HE staining results of the brain tissue (Figure 5) showed that the ultrasonic exposure combined with Sonovue^®^ did not damage the brain tissue, thus it is safe for the brain.

For Sonovue^®^ combined with Cytarabine of different dose groups, there was no brain tissue damage found in normal rats, and it was shown that there was not difference in the brain tissue between low-dose Cytarabine combined with ultrasound Sonovue^®^ group and high-dose ivdrip Cytarabine group (*P* < 0.05).

### The liquid chromatography-mass spectrometry (HPLC/MS) detection

**Figure 6:**
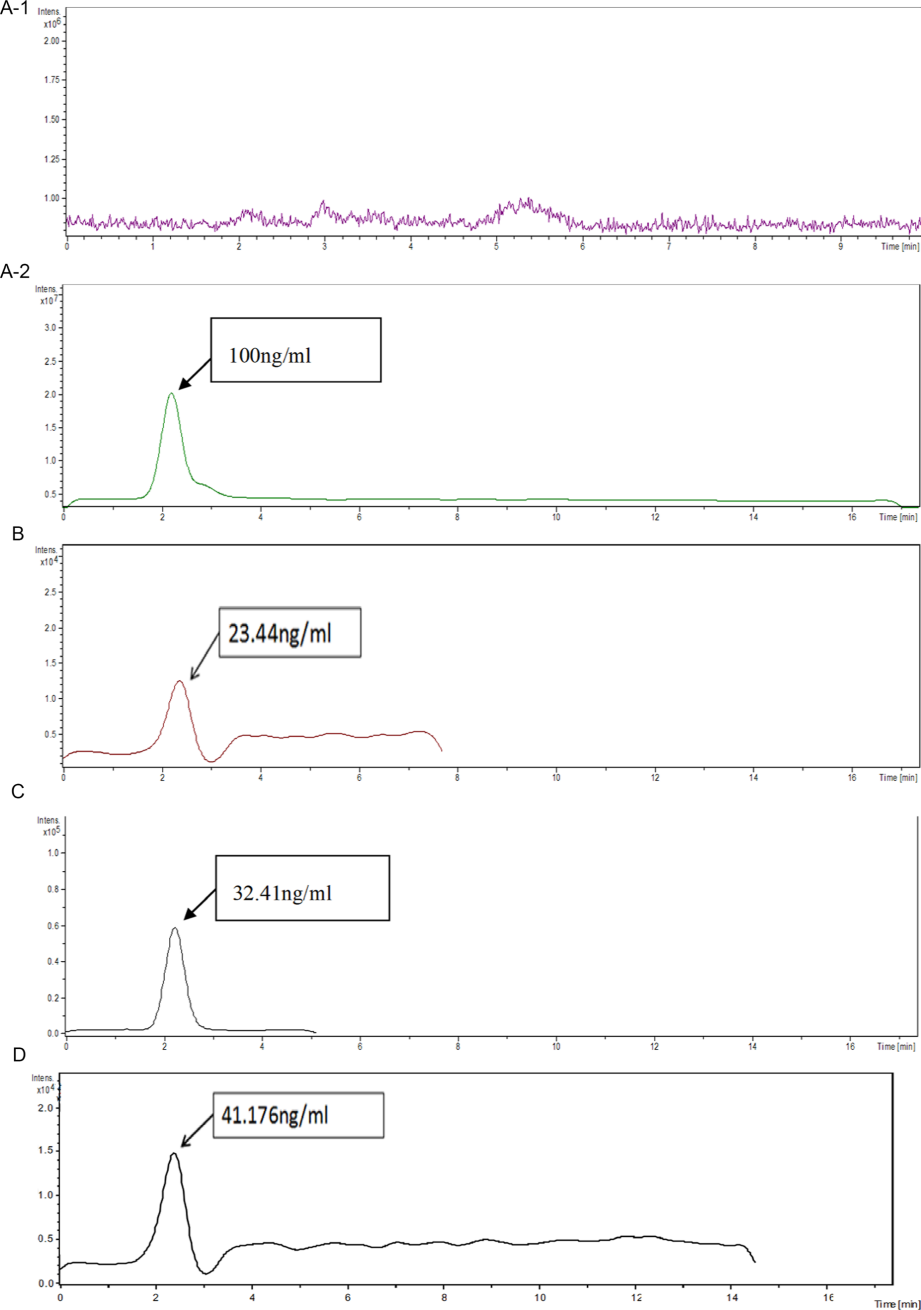
Brain tissue Cytarabine concentration measured by HPLC/MS under different groups: different groups and results corresponding with the results of Figure 3B (**A-1**) Blank Group; (**A-2**) Cytarabine standard ; (**B**) Low dose Group (10 mg/kg); (**C**) High dose Group (150 mg/kg); (**D**) Ultrasound Sonovue^®^ Drug Group (10 mg/kg).

HPLC/MS detection results listed in Table 3 and Figure 6A–6D showed that the Cytarabine concentration in rat brain of Group D is 41.176 ng/ml which is higher than that of high-dose Cytarabine group (Group C) of 32.41 ng/ml. Statistical analysis suggested there was no significant difference between group C and Group D (*P* > 0.05), however there was a statistically significant difference between low dose drug group (Group B) and Group D (*P* < 0.05). It showed that ultrasound combined with Sonovue^®^ can open the blood-brain barrier to promote drugs into the brain tissue to achieve effective treatment effect, and to reduce the side effects of chemotherapy that high-dose treatment is likely to bring.

**Table 3:** Ultrasound Sonovue^®^ after drug treatment group rats Cytarabine concentration (*n* = 4, $\bar{x}$ ± s)

group	Level	Dose Cytarabine injection	Cytarabine particle number	brain tissue Cytarabine concentration
A	Blank group	0	0	0
B	Low dose group	10 mg/kg	58713 ± 2.8	23.44 ng/ml
C	High dose group	150 mg/kg	81030 ± 8.6	32.41 ng/ml
D	Ultrasound combined with Sonovue^®^ and low dose group	10 mg/kg	102819 ± 5.0	41.18 ng/ml

## DISCUSSION

The presence of BBB makes it difficult for macromolecular chemotherapy drugs going through the blood-brain barrier and it has been a challenge to the treatment of CNSL [14]. Three treatment methods are clinically applied: more systemic high-dose chemotherapy, intrathecal injection and radiation therapy. These therapies can be used alone or combined. However, all the above three methods have certain disadvantages: high-dose chemotherapy could increase severe side effects; Intrathecal injection therapy is an invasive treatment with increased risk and low acceptance, especially lumbar puncture operation may cause damage to lumbar puncture (Traumatic lumber puncture, TLP) [15], and also the leukemia cells in the peripheral blood might infiltrate the cerebrospinal fluid due to operation and aggravate illness; Craniospinal radiotherapy has no selectivity and can cause brain damage.

Focused ultrasound (FUS) in conjunction with contrast agent microbubbles may involve delivery of systemically administered therapeutic agents to the CNS [12]. FUS and microbubbles induced targeted BBB may offer a solution to the problems associated with the delivery of drugs to the brain [5, 12, 16]. Several studies have shown that the duration of BBB depends on both the acoustic parameters and the concentrations of microbubble [17, 18]. Besides, it has been shown safe *in vitro* and *in vivo* [19, 20]. Recent studies indicated that low-intensity pulsed ultrasound could not only be proposed as a novel treatment modality for controllable release of drugs into the brain, but also ameliorate brain injury in the FUS-induced BBBD (BBB disruption) model [21]. The main purpose of this study was to prove that focused ultrasound combined with Sonovue^®^ can open the blood-brain barrier safely and especially at the same time can get the same drug concentration of brain tissue with intravenous injection of low-dose Cytarabine while it can be reached at the high- dose Cytarabine of intravenous drip. In our study, with HE staining method, it is proved that there is no obvious damage in brain tissue and blood vessels of the experimental group. Of course, we also can use above-mentioned ways to prove the same results furtherly. Nance *et al*. also demonstrate safe, pressure-dependent delivery of 60nm BPNs to the brain parenchyma in regions where the BBB is disrupted by FUS and MBs. They performed two MR-based safety studies on additional animals [22].

In the present study, our data validated that ultrasound exposure combined with Sonovue^®^ and Cytarabine can effectively promote the apoptosis of k562 cells via *in vivo* experiments at first. These results indicated that ultrasound exposure combined with Sonovue^®^ and Cytarabine may kill the leukemia cells which infiltrated in the central nervous system. Then it was shown that Ultrasound exposure combined with Sonovue^®^ could open the blood-brain barrier without brain tissue damage and effectively promote the drugs into the brain tissue via *in vitro* experiments. The Ara-c concentration within the brain tissue in low-dose Cytarabine combined with ultrasound and Sonovue^®^ can reach the concentration of that in high dose intravenous chemotherapy.

At very beginning we have been trying to culture the patient's chronic myelogenous leukemia cells which adopted from peripheral blood or bone marrow, even this method with human origin cells can be used to avoid the severe side effects that could be increased by high-dose chemotherapy, but we did not succeed because cell apoptosis developed quickly. We will try more research method. For *in vivo* experiments we used healthy Male Sprague-Dawley (SD) rats, based on the presumption that if normal blood brain barrier can be passed by Focused Ultrasound combined with drug and microbubble, the damaged blood brain barrier should be passed easier when the blood brain barrier (BBB) is destroyed at different levels by leukemia cells [23, 24]. Leukemia cells infiltrating tied to destroy blood brain barrier, blood-brain barrier damage further promote leukemia cells migrated to the central nervous system, which forms a vicious circle. Zhu *et al.* evaluate the clinical significance of circulating tight junction (TJ) proteins as biomarkers reflecting of leukaemia central nervous system (CNS) metastasis, TJs means claudin5 (CLDN5), occludin (OCLN) and ZO-1. The CNSL patients had a lower CLDN5/ZO1 ratio in both serum and CSF than in non-CNSL patients [25]. We will conduct further animal experiments to confirm these results in a leukemia animal model. If we can use the central nervous system leukemia animal model, the result will be more convincing. Li *et al.* successfully established a NOD/SCID mouse model of central nervous system leukemia by injection of acute monocytic leukemia cell line SHI-1 cells into the lateral ventricle. Brain tissue sections showed invasion into the subdural space, piamater, arachnoid, along the Virchow-Robin space and into the deep brain parenchyma [26]. We can learn from their practice of subsequent experiments.

Many studies have confirmed that ultrasound exposure combined with microbubble can target and open the blood-brain barrier reversibly and noninvasively, as well as make drugs, antibodies and genes infiltrate local brain tissue [27]. Although the specific mechanism is not clear, it provides a potential way for drug delivery through the blood-brain barrier and for the treatment of central nervous system diseases. This study provides a new way and an experiment foundation to treat central nervous system leukemia effectively.

## MATERIALS AND METHODS

### Cell culture

The k562 cells, a generous gift from Dr. JI, were cultured in RPMI1640 (Hyclone) containing 10% FBS (Hyclone) and 1% penicillin/streptomycin (Gibco) and maintained in a humidified incubator at 37°C (Thermo Fisher Scientific Co., Ltd., Shanghai, China) subculturing every 2 to 3 days. The logarithmic growth cells were selected for the experiment.

### Sonovue^®^ (White freeze-dried powder, Bracco, Italy)

Sulfur hexafluoride microbubbles for injection, with a bottle containing sulfur hexafluoride gas: 59 mg, freeze-dried powder: 25 mg. When used with 5 ml saline, form microbubbles mixed suspension after shaking, Sonovue^®^ concentration is 16.8 mg/ml, the average particle size is 2.5 um, microbubble concentration is 1–5 × 10^8^/ml. Room temperature preservation of six hours.

### Cytarabine preparation

Cytarabine (for Injection, Pharmacia Italia S.P.A), mix with 5 ml diluents (include 45 mg benzyl alcohol), compound the mother liquor with final concentration of 20 mg/ml and save in –20°C refrigerator.

### Ultrasound exposure

The operating frequency of ultrasonic instrument (Beijing Dongjian Company) is 800 KHz, ultrasonic probe area is 2.8 cm^2^. The ultrasound wave was generated in the pulse mode with the pulse repetition frequency of 50 Hz. Other adjustable parameters include sound intensity, duty ratio and time. Duty ratio refers to the pulse signal of the current time and the ratio of the current cycle: In a series of pulse cycle sequence, the pulse duration and pulse cycle ratio. Orthogonal experimental design and extreme difference analysis were used to choose the optimal experimental parameters.

### Annexin V - FITC/PI flow cytometry double marking method to test cell apoptosis rate

The k562 cells were collected with PBS liquid and washed twice, took about 5 **×** 10^5^ in the concentration of cell suspension, centrifuge at 2000 RPM 5 min, to join 500 ml Binding Buffer cells suspended: add 5 ul Annexin V-FITC blender, add 5 ul PI (propidium lodide), gently blending; at room temperature, avoid light, react 5 to 15 min. Use flow cytometry for testing within 1 hour. At the same time use k562 cells without Annexin V-FITC/PI cells for debugging. This experiment was repeated three times.

### Animals

Male Sprague-Dawley (SD) rats weighing from 280 to 300 g were used in this study. Before ultrasound exposure, each animal was anesthetized in the prone position by inhalation of 2% isoflurane in 2 l/min oxygen, and the body temperature was maintained at 37°C using a heating pad. The top of the rat cranium was shaved for ultrasound exposure.

Ultrasonic Parameters: using an ultrasonic frequency of 800 KHz's commoditized therapeutic ultrasound (China Dongjian Company), the manual probe area is 2.8 cm^2^. Ultrasonic parameter selection: pulse mode, pulse repetition frequency 50Hz, ultrasound intensity: 2.56 W/cm^2^, the duty ratio: 1:1, duration time: 5 min.

The ultrasonic coupling agent was applied at the top of the cranium, and the probe was pointed at the junction between the two ears of the rat and the binocular line, and the ultrasonic continuity pathway was established.

The small dose group was given 10 mg/kg, large dose was given 150 mg/kg, followed by a 0.01 ml/100g dose. Injection Sonovue^®^ within 30 seconds after injection of Cytarabine, ultrasonic parameter selection: pulse mode, pulse repetition frequency 50 Hz, ultrasound intensity: 2.56 W/cm2, the duty ratio: 1:1, time: 5 min. The left atrium perfusion fixation: cut open thoracic, visible actual beating heart, the left cardiac apex and insert needle with hemostatic forceps firmly, cut right atrium, with 50 ml/min speed drops into the 100–200 ml, 0.9% saline water injected another 300–350 ml of 4% paraformaldehyde fixed liquid. The brain tissues were taken and HE stained for routine microscopic evaluation.

### HPLC/MS was used to measure the concentration of Cytarabine in the brain

Weigh and record the brain tissue of each group. Add 1 ml physiological saline per gram tissue. Measuring cerebral tissue homogenizes 0.1 ml, followed by adding 7% perchloric acid 0.2 ml, and oscillated and mixed 2 minutes to precipitate the protein, centrifuge at 10,000 r/min for 5 minutes. Take 20 ul clear liquid from the bottom of the glass tube tip, into the automatic sampler of the high performance liquid chromatography-mass spectrometer (HPLC-MS, Agilent 1100 HPLC system coupled with a LC-MSD Trap SL MS detector, Agilent MA USA) operated at SRM mode (m/z:244.0 → 112.1). The mobile phase was a mixture of methanol and 1% formic acid solution (70:30, v:v), a Zorbax SB C18 column was utilized at a flow rate of 0.8 ml/min, and a 0.2 ml/min portion of the elute was delivered to the ESI ion source. Use 20 ul sample and then figure out, record the chromatogram and peak area. In addition, the standard samples of Cytarabine were also recorded, and the chromatogram and peak area were recorded.

### Statistical analysis

All values are shown as means + SEM. Statistical analysis was performed using an unpaired Student *t* test. The level of statistical significance was set at *P* < 0.05.
